# Credibility Enhancing Displays, religious scandal and the decline of Irish Catholic orthodoxy

**DOI:** 10.1017/ehs.2022.21

**Published:** 2022-05-25

**Authors:** Hugh D. Turpin, Aiyana K. Willard

**Affiliations:** 1 Centre for the Study of Social Cohesion, School of Anthropology and Museum Ethnography, University of Oxford, Oxford, UK; 2 Centre for Culture and Evolution, Division of Psychology, Department of Life Sciences, Brunel University, London, UK

**Keywords:** Credibility Enhancing Displays, Irish Catholicism, secularisation, clerical abuse, religious hypocrisy, nonreligion

## Abstract

Credibility Enhancing Displays have been shown to be an important component in the transmission of empirically unverifiable cultural content such as religious beliefs. Decreased Credibility Enhancing Displays are a major predictor of religious decline. However, because declines in belief are often paired with the decreasing importance of religious institutions, existing research has not yet shown the effect of Credibility Enhancing Displays as separate from this institutional decline. Here, we assess the role of past Credibility Enhancing Display exposure among the baptised Catholic population of Ireland in predicting who retains a Catholic identity and religious beliefs among those who reject the Catholic Church. We find that leaving Catholicism outright (i.e. ‘ex-Catholicism’) is predicted by low Credibility Enhancing Display exposure, but rejecting the Church while retaining a Catholic identity (i.e. ‘liminal Catholicism’) and theistic belief is not. High perceived prevalence of clerical paedophiles (i.e. religious hypocrisy) predicts both groups similarly. Higher exposure to Credibility Enhancing Displays predicts higher orthodox Catholic beliefs and Catholic morality among Catholics, but with inconsistent and even negative effects among the other groups. High perceived prevalence of clerical paedophiles predicts the rejection of orthodox Catholic beliefs, but not the rejection of theism or a Catholic identity.

## Graphical abstract

**Figure:**
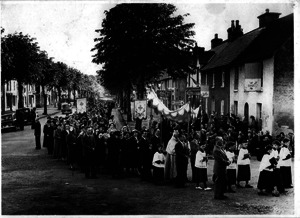

**Social media summary:** High estimates of clerical paedophilia predict the rejection of orthodox tenets of belief among Irish individuals who have been baptised Catholic, but only low levels of religious signalling from parents predict leaving Catholicism entirely and rejecting theism in general.

## 1. Introduction

Cultural evolutionary research has suggested that people are more likely to accept empirically unverifiable beliefs (for instance the belief that there is a God, or that He is Three Divine Persons in One) if they have witnessed other people's behavioural commitment to these same beliefs (Henrich, 2009). These behavioural cues are known as ‘Credibility Enhancing Displays’. Credibility Enhancing Displays consist of ‘costly’ or ‘hard to fake’ signals such as ritual participation, taboo observation, scarification, celibacy and others. Such acts offer proof that belief is more than a matter of lip service and should be taken seriously by cultural learners (Lanman & Buhrmeister, 2016). When religious beliefs fail to transmit across generations in large swathes of society, it may be because social changes have precipitated a decline in supporting Credibility Enhancing Displays (Lanman, 2012; Willard & Cingl, 2017). In essence, while people may still believe in some religious propositions, they stop participating in the ritual and other behavioural practices necessary to instil these beliefs in the next generation (Gervais, Najle & Caluori, 2021). The result is that religious beliefs become peripheral add-ons rather than core components of shared in-group worldviews, and they are more easily neglected or rejected by cultural learners. Over the space of a generation religious belief begins to fade away.

One critique of existing empirical literature on Credibility Enhancing Displays is the difficulty in separating specific religious beliefs, identities and values from an affiliation with a religious institution. Those exposed to higher Credibility Enhancing Displays tend to report maintaining high levels of religious belief and identity, but the literature thus far has not been very specific on what this actually entails (e.g. Willard & Cingl, 2017). Religiosity declines with declines in exposure to Credibility Enhancing Displays, but also with the decline in importance of religious institutions and all the things that go with them. This makes it difficult to determine the effect of behavioural displays on belief itself. We can sperate this in a sample where an institution is rejected for a reason other than lack of belief – in this case the rejection of the Catholic Church owing to clerical abuse scandals in Ireland – by testing if Credibility Enhancing Displays predict the maintenance of religiosity even when the religious institution itself has been rejected.

Here, we assess the role of past Credibility Enhancing Display exposure among the baptised Catholic population of Ireland in predicting who retains and who rejects orthodox Catholic religious beliefs and Catholic identity in a time of widespread church scandal. Many who have rejected the authority of the Catholic Church have also rejected associated doctrinal beliefs, but not all have. What makes this a particularly interesting setting for furthering our understanding of Credibility Enhancing Displays is that moral scandal gives people a reason to reject Catholicism that is unrelated to exposure to the sorts of ritual behaviours that are thought to instil long-lasting belief. Here, we test if past Credibility Enhancing Display exposure can help us differentiate those who maintain some levels of religious belief from those who do not within the broader group who have rejected the authority of the Church. We also look at the role of Credibility Enhancing Display exposure in determining the acceptance of theism in general, specific orthodox Catholic theological propositions (e.g. the Virgin Birth or the Trinity) and symbolic divine moral edicts associated with the Church (e.g. opposition to contraception and abortion).

In addition to the predictive role of past Credibility Enhancing Display exposure in reinforcing Catholicism, we also examine the link between clerical scandal and the rejection of Catholicism. Outrage at religious hypocrisy – the very opposite of a Credibility Enhancing Display – has been a highly salient feature of Irish public discourse for three decades. Ireland's prolific clerical abuse scandals could be taken to indicate that the Church very much does *not* believe in the morally interested God it verbally endorses, nor in the strict moral codes it once enforced on others with such zeal (Hilliard, 2003; Turpin et al., 2017). Since the late 1990s, trust in the Church has declined more quickly in Ireland than in other European Catholic societies (Donnelly & Inglis, 2010). For many, the institutional Church has become an object of contempt (Iona Institute, 2011; Inglis, 2014; Scally, 2021; Turpin, 2022). Nevertheless, while most of the population have been shocked by these scandals, not all have renounced Catholicism as a result (e.g. Ganiel, 2016, 2019). Against this cultural backdrop, we hypothesise that lower domestic Credibility Enhancing Display exposure and higher perceived prevalence of clerical paedophilia combine to significantly undermine Catholic belief and to predict decisions to leave Catholicism. We predict that lower Credibility Enhancing Display exposure will differentiate those who have left the religion entirely (i.e. ‘ex-Catholics’) from those who have remained, but that past Credibility Enhancing Display exposure will not differentiate between orthodox Catholics and those who have retained a Catholic identity but rejected the authority of the Church (‘liminal Catholics’). We predict that higher perceived prevalence of clerical paedophilia will predict membership in both groups of leavers compared with orthodox Catholics.

As well as looking at theism, we take a more exploratory approach to examining other beliefs and morals central to Catholicism. Since these beliefs are more specifically related to Church authority than belief in God, we expect that both groups of leavers will reject these to some degree. We will test the role that Credibility Enhancing Displays and perceived prevalence of clerical paedophilia play in commitment to these beliefs and moral values across all three groups.

### 1.1. Credibility Enhancing Displays

Recent work examining the adoption of religious beliefs has emphasised the role of *context biases* in their transmission (Gervais, Willard, Norenzayan, A., & Henrich, 2011; Gervais et al., 2021). These are evolved tendencies to selectively accept information based on contextual social cues such as the prestige, competence or conformity of the information source (Boyd, Richerson & Henrich, 2011; Henrich, 2015). One bias with particular importance for religious transmission is thought to be the ‘Credibility Enhancing Display’ bias (Henrich, 2009). The theory of Credibility Enhancing Displays argues that humans use a learning heuristic to avoid deception by preferentially attending to verbal statements backed up by hard to fake behavioural evidence of true belief. For example, people are more likely to adopt a positive view towards, and install, solar panels when they see that those who endorse solar energy also install their own panels (Kraft-Todd et al., 2018). Religions that have culturally evolved to exploit this bias by associating beliefs with costly or hard to fake behaviours should become more successful (Henrich, 2009).

Current evidence for the role of such displays in belief transmission is promising if somewhat sparse. Henrich's original 2009 paper combined an analytic model with supporting evidence from developmental and social psychological sources (e.g. Harper & Sanders, 1975; Walster, Aronson & Abrahams, 1966; Bryan & Walbeck, 1970; Grusec, 1971; Harris, Pasquini, Duke, Asscher, & Pons, 2006). Subsequently, Lanman and Buhrmeister (2017) created and used a ‘Credibility Enhancing Display scale’ to find that parental exposure to these behaviours was the strongest predictor of whether or not an individual became a theist or non-theist. Willard and Cingl (2017) found that differing levels of childhood exposure to Credibility Enhancing Displays were a strong predictor of the large differences in theism between otherwise culturally similar Czechs and Slovaks. Gervais et al. (2021) similarly found that low Credibility Enhancing Display exposure was the strongest predictor of religious decline in a representative sample from the US. Using their own correlational measure and a large sample of 67,000 individuals, Maij and colleagues (2017) argued that exposure to Credibility Enhancing Displays plays a much larger role in generating religious belief than individual differences in sensitivity to content features. Langston and colleagues also found that higher exposure predicted a later age of religious rejection among members of online New Atheist communities (Langston, Speed, & Coleman, 2020). While most studies have been correlational, Willard and colleagues found that people were more amenable to believing counterintuitive scientific information if they saw an experimental confederate performing a supporting Credibility Enhancing Display for the information in question (Willard, Henrich, & Norenzayan, 2016).

At a theoretical level, such religious displays have also been tied to the process of secularisation. Sociological data suggest that intergenerational declines in belief tend to occur when existential security rises (Norris & Inglehart, 2011; Inglehart, 2021). Lanman proposes that declines in Credibility Enhancing Displays underlie this causal relationship (Lanman, 2012). According to Lanman, religious beliefs act as identity markers which signal in-group commitment (Sosis, Kress & Boster, 2007; Henrich, 2009; Atran, 2010). Under conditions of threat, commitment to shared in-group beliefs and values escalates, probably as a means of recruiting coalitional support (Greenberg, Solomon, & Pyszczynski, 1997; Navarrete & Fessler, 2005; Eriksen, Bal, & Salemink, 2010). These dynamics increase the prevalence of public actions, indicating commitment to shared (frequently religious) worldviews, and this bolsters the transmission of associated religious beliefs for cultural learners. As threat declines, so does religious action. Lanman argues that his ‘threat and action’ theory can help explain differing international levels of religious belief (for example, low levels in existentially secure Scandinavia and higher levels in the more existentially precarious USA). However, more work is needed to firmly tie declines in Credibility Enhancing Displays to declining religious belief (including which particular beliefs are declining), and to the social changes behind the process of secularisation.

### 1.2. Irish secularisation

Ireland offers a particularly interesting environment to examine the relationship between Credibility Enhancing Displays and secularisation. Until the 1990s, Ireland was one of the most impoverished societies in Western Europe – and one of the most religious (Garvin, 2004). During the nineteenth century, under the influence of Cardinal Cullen, Irish Catholic orthodoxy was amplified in the wake of the Great Famine, developing into a highly self-abnegating and performative style of devout obeisance based on tight adherence to Roman best practice (Larkin, 1976; Inglis, 1998; Scally, 2021). From a cultural evolutionary perspective, costly religious signalling proliferated wildly. One scholar has described the behavioural adornments of post-Famine Catholicism as follows:

Confession and communion … became much more frequent. Pastoral gains … were consolidated by the introduction of a whole series of devotional exercises designed not only to encourage more frequent participation in the sacraments but to instill veneration by an appreciation of their ritual beauty and intrinsic mystery … The new devotions were mainly of Roman origin and included the rosary, forty hours, perpetual adoration, novenas, blessed altars, Via Crucis, benediction, vespers, devotion to the Sacred Heart and to the Immaculate Conception, jubilees, triduums, pilgrimages, shrines, processions, and retreats. (Larkin, 1976: 77)

This highly behaviourally enriched religious culture persisted deep into the twentieth century. After independence in 1922, Church and State became very closely intertwined. Poverty, nationalism and integralism meant Catholicism continued to play a central role in Irish life and group identity. Both public and domestic life were saturated with religious signalling: to be Irish was to be (and to be seen to be) a ‘good Catholic’ (Inglis, 1998). Indeed, the traditional ‘Irish Catholic mother’ was memorably described by the influential sociologist Tom Inglis as ‘the living embodiment of Our Lady – humble, pious, celibate and yet fecund’ (Inglis, 1998: 249). Alongside the public dominion of the Church, Inglis described a culture of parallel domestic religious reinforcement, with mothers acting as ‘the Church's representative in the home’ (Inglis, 1998). Stemming in part from this culture, survey data has long indicated that religious affiliation and belief have remained unusually high in Ireland compared with elsewhere in Western Europe (e.g. Bullivant, 2017).

Nevertheless, recent decades have seen a rapid shift towards secularisation. Owing to top-down shifts in economic and social policy and bottom-up processes of cultural globalisation, Catholic isolationism declined from the 1960s onwards (Inglis, 1998; O'Toole, 2021). From the 2000s onwards, Irish religious and moral attitudes realigned with those more typical of other European societies (Fuller, 2002; Penet, 2008). In particular, the ‘Celtic Tiger’ economic boom of the 1990s and 2000s precipitated a ‘change in people's perceptions of themselves, their lifestyles, morale, confidence and sense of independence’ (Fuller, 2002: 232). Religious attendance collapsed over this period (Donnelly & Inglis, 2010). While the last Irish Census in 2016 recorded 78.3% of the population as Catholic, the nature of the link has changed greatly. Sociologists describe how orthodox piety has largely come to be replaced by low-cost ‘cultural Catholicism’, revolving around a small number of culturally important and lightly enjoyable rites of passage (Inglis, 1998; McWilliams, 2019). Yet while ‘cultural religion’ has prevailed, Credibility Enhancing Displays have collapsed. In the words of one observer, Ireland is now an increasingly ‘ambivalent place’ where growing numbers ‘don't believe a word of it’ but baptise their children anyway, largely for reasons of belonging, social expedience and cultural habit rather than out of any sense of piety (McWilliams, 2019: 2). Religion, for the most part, is undemanding and quarantined to rare occasions.

This low-cost version of Catholicism may not mark a stable equilibrium. The last decade has seen the emergence and rapid growth of unaffiliated Irish people. In censuses, the religiously unaffiliated rose from 4% in 2002 to 10% in 2016. In the European Social Survey, a measure less prone to ethno-nationally motivated nominal affiliation, non-religion rose from 14% in 2002 to 32% in 2018. According to Win-Gallup (2012), Ireland now has a greater proportion of ‘convinced atheists’ than Britain, Germany and the Scandinavian countries. The Credibility Enhancing Display-poor culturally Catholic system does not appear to be producing many committed believers – and, in combination with clerical scandals and the Church's perceived anti-progressive influence, it may even be generating a considerable amount of anti-theism, with Irish non-religious individuals by some measures the most anti-religious in Western Europe (Ribberink, Achterberg, & Houtman, 2013).

### 1.3. Clerical abuse scandals and religious hypocrisy

One factor which complicates any clear relationship between Credibility Enhancing Display collapse and rising Irish religious rejection is the growth in public perceptions of religious hypocrisy on the part of Catholic paragons. Ireland's period of social liberalisation and economic boom coincided with the moral discrediting of the Church. Those socialised in the period from the 1990s onwards were not simply exposed to lower religious behavioural signalling; they were also ‘born into a Church of scandal’, as one clerical informant described it (Turpin, 2022). ‘The scandals’ commenced in the early 1990s with national news reports of violated clerical chastity, escalating swiftly to reports of child abuse and cover-ups. From there, the scandals widened to include the reappraisal of coercive carceral institutions operated by Catholic orders, such as Industrial Schools, Magdalene Laundries, and Mother and Baby Homes (Finnegan, 2001; Raftery & O'Sullivan, 2009; Hogan, 2019). The Church and its orders are now frequently represented as immoral forces to be opposed rather than social paragons to be emulated (e.g. Pine, 2011). Clerical abuse is commonly framed as a form of extreme hypocrisy, given the Church's historical interest in dictating sexual behaviour and punishing deviation from strict conservative norms (Goode, McGee, & O'Boyle, 2003; Hilliard, 2003; Hogan, 2019). One 2003 study on Irish public responses to the clerical abuse scandals described a public who were ‘horrified’ not only ‘because people abhor child sexual abuse *per se*’ but also because abuse constituted ‘the polar opposite of what priests and religious are meant to be doing’ (Goode et al., 2003).

Today, the Irish association between the Catholic Church and the arch-crime of paedophilia is particularly deeply entrenched (e.g. Goode et al., 2003; Angelides, 2005; Keenan, 2012). According to a survey by a conservative Catholic think tank, the average population estimate of the number of clergy who are paedophiles is 28%, with 42% of the population advancing estimates in excess of 21% (Iona Institute, 2011). This same survey also found that almost a third of the Irish Catholic population would not be concerned if the Church ceased to exist. As a result of clerical abuse scandals, while individual priests may still be evaluated as ‘good people’, the clerical category as a whole has become a target of much popular contempt (Goode et al., 2003; Egan, 2011; Turpin, 2022). From a context-based learning perspective, the contemptible are the opposite of the prestigious; they are probably the very last people a social learner would seek to learn from (e.g. Gervais & Fessler, 2017). This may be particularly true of those deemed contemptible specifically because of their perceived hypocrisy. Hypocrisy is processed as the ultimate warning signal of the deceptive free rider (Jordan, Sommers, Bloom, & Rand, 2017). Such paragon hypocrisy may be particularly undermining for religious beliefs, because their acceptance is more dependent on trust and social proof than more directly verifiable propositions (e.g. Shupe, 1997; Wollschleger & Beach, 2011). The social category of priests, in other words, has become a prominent source of ‘Credibility *Undermining* Displays’ for Catholic representations (e.g. Turpin et al., 2017; Turpin, 2019). Priests probably hold this status in the eyes of disengaged Catholics in particular, as their attitudes towards clergy are liable to be informed more by national media than by personal exposure to more positive exemplars (e.g. Donnelly & Inglis, 2010). In other words, a foundation of low Credibility Enhancing Display exposure probably predisposes somebody to be more influenced by media-relayed clerical Credibility Undermining Displays.

Here, we examine how past exposure to parental Credibility Enhancing Displays and current contempt for clergy relate to different religious outcomes among contemporary Irish baptised Catholics. We examine how these variables relate to three dimensions of ‘being Catholic’: Catholic affiliation, religious belief (both in God generally and in specific Catholic shibboleths such as transubstantiation) and acceptance of Catholic moral edicts. The data illuminate the role of past exposure to Credibility Enhancing Displays and present disdain for the Church in predicting those who renounce or maintain belief in God, those who renounce Catholic affiliation and those who do not, and those who reject symbolic Catholic beliefs and edicts and those who do not.

## 2. Methods

The current data are from an online survey run in 2017 (Turpin, 2022). The sample comprised 247 Irish individuals of 18 years of age and over who had been baptised Catholic. This sample was acquired via Qualtrics Panels and was nationally representative for age, gender and socioeconomic status (as represented by standardised categories regarding the occupation of the chief income earner in the household). The survey also contained a representative mix of urban and rural respondents (26% described themselves as living in a city, 13% a suburb, 25% a town, 12% a village and 24% the countryside: a fairly even breakdown of Irish habitation). This variable was included as rural Ireland is often thought to be more religious than the major urban centres, but owing to a number of participants neglecting to answer the question, it was not included in the analyses below. Respondents were also asked whether they had been educated in Catholic schools. Of the 204 participants who answered this question, 163 had been educated at both primary and secondary level in Catholic schools, 24 had been educated in a Catholic school at primary level only, 17 at secondary only and only 1 participant had received no Catholic schooling at all. Unfortunately we did not record education level among our demographics. The survey had a high response rate of 45%.

### 2.1. Establishing Catholic, liminal Catholic and ex-Catholic groups

Respondents were first asked whether they were baptised Catholics (‘Have you been baptised as a Catholic?’). ‘Natalism’ is thought to inflate Irish Catholic statistics by eliciting affiliative responses based on past baptismal status rather than current rejection of Catholic belief or membership. By asking participants if they had been baptised first and establishing that participants had been ‘made Catholic’, we aimed to diminish ethno-natalist responding in the remainder of the survey (e.g. Day, 2011). The survey then adopted an alternative two-step approach to measuring the current commitment of an individual to Catholicism, based around (a) self-designation (i.e. affiliation) and (b) self-perceived acceptance of doctrine (i.e. belief). Firstly, participants were asked how they currently described themselves (‘Would you describe yourself as any of these? Please select all that apply – you may choose more than one if you wish: Agnostic; Atheist; Catholic; Christian – no denomination; Member of a non-Christian religion; Protestant Christian – any denomination; Spiritual but not religious; None of these’). After this, participants were asked whether they rejected the ‘official’ doctrines and teachings of the Catholic Church (‘Overall, would you say you personally have or have not rejected the official beliefs, doctrines and teachings of the Catholic Church as guidelines for your life?’). This procedure allowed the rejection of Catholicism to be characterised with respect to two components: the rejection of a Catholic identity (e.g. disaffiliation) and the rejection of Church teachings (disbelief in institutional orthodoxies). It is possible to reject either, both or neither. This leaves the three larger groupings: Catholics, liminal Catholics and ex-Catholics. Catholics were operationalised as those who identify as Catholic and do not reject the teachings of the Church, liminal Catholics as those who identify as Catholic but reject the teachings of the Church and ex-Catholics as those who reject both a Catholic identity and the teachings of the Church. The designation ‘ex-Catholic’ can be justified by the degree to which religious and ethnic identity are essentially fused in an Irish context. Whether one is religious or not, once baptised, ‘being Catholic’ is something one just ‘is’. This is reflected in the tendency of many Irish people, including those utterly disinterested in religion, to describe themselves as having been ‘born Catholic’, even if they do not believe. Ex-Catholicism represents self-conscious exit not just from religion as a worldview, but from an ethno-religious status ascribed in infancy. It represents a decision to opt out. The data reflect this in the ages liminal and ex-Catholics report rejecting the authority of the Church: mean liminal rejection occurs at 21 years of age, while mean ex-Catholic rejection of the Church occurs at 18 years of age. There were also nine people who rejected a Catholic identity but did not reject the teachings of the Church. Owing to their rejection of a Catholic identity, these participants were deemed most appropriate to be included in the ex-Catholic category. Overall, this yielded 97 Catholics (39.3%), 60 liminal Catholics (24.3%) and 90 ex-Catholics (36.4%).

### 2.2. Scale measures: orthodox Catholic belief, theism, Church moral agreement and Credibility Enhancing Display exposure

Theism was measured using a Likert scale ranging from 1 (certain that God does not exist) to 7 (certain that God exists). Commitment to Catholic religious beliefs was assessed using an orthodox Catholic belief scale (seven-point Likert, *α* = 0.96, six items: transubstantiation, papal infallibility, the Resurrection, miracles, the Virgin Birth, Confession). Adherence to orthodox Catholic moral positions was measured using a Catholic moral agreement scale which asked participants to rate how much they concurred with the Church's official positions on a number of key moral questions (seven-point Likert, *α* = 0.89, eight items: abortion, same-sex marriage, divorce, sex before marriage, priestly celibacy, female ordination, and contraception). Credibility Enhancing Display exposure was measured using a Catholic version of Lanman and Buhrmester's 2017 Credibility Enhancing Display scale (seven-point Likert, *α* = 0.92, 8 items). These scales can be viewed in Appendix I.

### 2.3. Perceived prevalence of clerical paedophiles as a proxy for moral contempt for the Catholic Church

Participants were also asked to estimate the percentage of Catholic priests who had abused children. These estimates are used below as a proxy for moral contempt for the Catholic Church. Orthodox Catholics gave a mean estimate that 20.3% of priests were paedophiles. The liminal Catholic mean estimation was 36.1%. The ex-Catholic mean estimation was 39.5%.

### 2.4. Analyses

Alongside demographics, Credibility Enhancing Display exposure and the clerical contempt measure were entered into a multinomial logistic regression model to predict membership in the Catholic, liminal Catholic and ex-Catholic groups, as well as three linear regression models to predict levels of Catholic belief, levels of general theism and acceptance of orthodox moral edicts. Continuous variables, other than age and percentage estimates of clerical paedophilia, were standardised before analysis. We hypothesised that lower Credibility Enhancing Display exposure would predict becoming an ex-Catholic, but not a liminal Catholic, and that liminal Catholics would maintain theistic belief where ex-Catholics would not. We also hypothesised that lower Credibility Enhancing Display exposure and higher clerical contempt would predict rejection of orthodox Catholic belief and orthodox Catholic morality.

## 3. Results

### 3.1. Credibility Enhancing Displays, moral contempt and the rejection of Catholic affiliation

Can past Credibility Enhancing Display exposure predict who becomes an ex-Catholic (i.e. rejects Catholicism entirely), who becomes a liminal Catholic (i.e. retains Catholic affiliation while rejecting the authority of the Church) and who remains an obedient Catholic? Is the effect of Credibility Enhancing Displays separate from the belief that priests are paedophiles?

**Figure 1.**
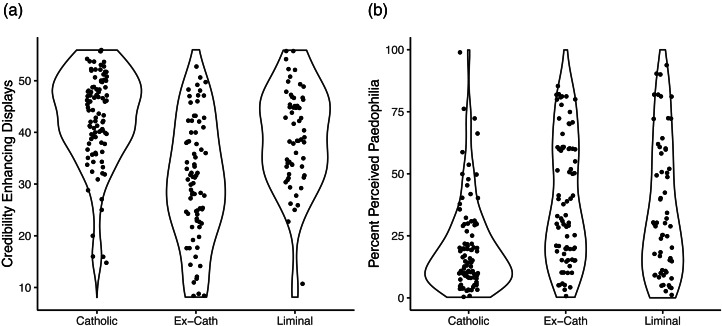
The distribution of Credibility Enhancing Display scores (a) and predicted percentages of Catholic clergy who are paedophiles (b) in the Catholic, ex-Catholic and liminal Catholic groups.

Initial descriptive statistics found that the groups differed on Credibility Enhancing Displays in the expected way. Catholics had a mean Credibility Enhancing Display score of 5.39 (SD = 1.14), liminal Catholics had a mean score of 4.88 (SD = 1.27) and ex-Catholics had a mean score of 3.81 (SD = 1.51). The distribution of Credibility Enhancing Display scores and estimates of paedophilia in the clergy within each group can be inspected in Figure 1. It can be visually conformed that individuals with very low Credibility Enhancing Display scores tend to be found most frequently in the ex-Catholic group. The correlation between Credibility Enhancing Displays and estimates of paedophilia is −0.21 (95% CI, −0.33 to −0.09). Age significantly predicted exposure to Credibility Enhancing Displays (*ß* = 0.29, 95% CI = 0.22–0.36) and women had marginally higher scores than men (*ß* = 0.21, 95% CI = −0.01–0.44). Age was a marginal negative predictor of estimates of paedophilia (*ß* = −0.16, 95% CI = −0.36–0.03), and women gave higher estimates than men (*ß* = 0.86, 95% CI = 0.23–1.50).

We next ran a multinomial logistic regression model predicting group membership. Catholic membership was the reference category (Table 1). Model 1 used only Credibility Enhancing Displays and demographic variables as predictors. As a proxy for moral contempt, model 2 included the predicted percentage of priests who were paedophiles.

**Table 1.** Multinomial models predicting the effect of demographic factors, Credibility Enhancing Displays and moral contempt for the Church on Catholic group membership

		Model 1		Model 2
Predictors	Odds ratios	95% CI	Odds ratios	95% CI
*Ex-Catholic*				
(Intercept)	1.16	0.73–1.83	1.60	0.95–2.68
CREDs	0.32*	0.21–0.49	0.35*	0.22–0.55
Age	0.77*	0.61–0.97	0.74*	0.57–0.94
Gender (F)	0.44*	0.22–0.87	0.28*	0.13–0.60
% Paedo			1.48*	1.25–1.75
*Liminal Catholic*				
(Intercept)	0.59*	0.35–0.99	0.82	0.47–1.46
CREDs	0.71	0.45–1.10	0.73	0.46–1.17
Age	0.82	0.65–1.03	0.82	0.64–1.04
Gender (F)	1.25	0.63–2.46	0.84	0.40–1.74
% Paedo			1.38*	1.17–1.62

*Note:* *confidence interval does not cross 1.

CRED, Credibility Enhancing Display.

**Figure 2.**
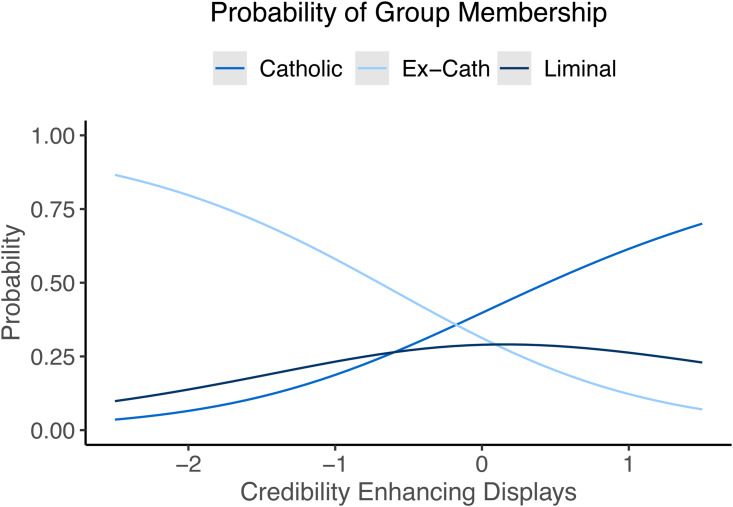
Probability of Credibility Enhancing Displays predicting membership in Catholic, ex-Catholic or liminal Catholic groups.

Exposure to Credibility Enhancing Displays was related to a decreased odds of being an ex-Catholic (i.e. this group was exposed to lower Credibility Enhancing Displays, on average) in these models (Figure 2). Exposure to Credibility Enhancing Displays predicted a smaller decrease in the odds of being a liminal Catholic, but this effect is not significant in any of the models. Belief that there are more paedophiles in the Church was related to an increased odds of being an ex-Catholic or a liminal Catholic.

### 3.2. Theism

**Figure 3.**
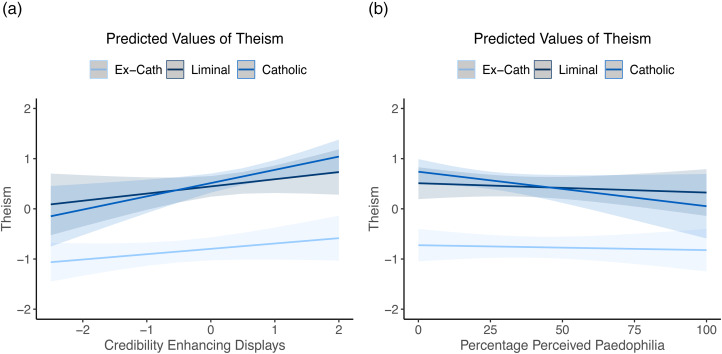
Linear relationships between (a) Credibility Enhancing Display exposure and theism, and (b) perceived clerical paedophilia and theism. Shaded areas are 95% confidence intervals.

Across our three groups, belief in God only showed clear signs of decline among ex-Catholics. When asked if they believe in God (yes/no) 98.97% of the Catholics, 100% of the liminal Catholics and 23.33% of the ex-Catholics said yes. Similar results were found when participants were asked about their degree of belief (1–7). Ex-Catholics (mean = 2.72, SD = 1.75) rated their belief both sizably and significantly lower than Catholics (mean = 5.92, SD = 1.43), but liminal Catholics did not (mean = 5.53, SD = 1.19). To look at group level differences in belief we first ran a regression model using demographic factors alone (model 1, Table 2). Credibility Enhancing Displays and predictions about paedophilia were added to model 2, as was an interaction effect between groups and Credibility Enhancing Displays. Model 3 replaces this interaction with an interaction between group and paedophilia predictions. These interactions are plotted in Figure 3. Credibility Enhancing Display scores predicted belief overall (*ß* = 0.26, 95% CI = 0.01–0.28; Table 2), with a slightly but not significantly weaker effect for liminal (simple slope, *ß* = 0.14, 95% CI = −0.08–0.36) and ex-Catholics (simple slope, *ß* = 0.11, 95% CI = −0.03–0.26). Estimates of the percentage of paedophiles in the church did not predict theistic belief (simple slopes – Catholic, *ß* = −0.07, 95% CI = −0.15–0.01; liminal, *ß* = −0.02, 95% CI = −0.08–0.05; ex-Catholic, *ß* = −0.03, 95% CI = −0.08–0.03).

**Table 2.** Strength of belief in God is predicted by past Credibility Enhancing Display exposure but not by perceived prevalence of clerical paedophiles

	Model 1		Model 2		Model 3	
Predictors	Estimates	95% CI	Estimates	95% CI	Estimates	95% CI
(Intercept)	0.32	−0.12–0.75	0.32	−0.12–0.77	0.27	−0.19–0.73
Ex-Cath	−1.48*	−1.70 to −1.26	−1.29*	−1.54 to −1.04	−1.27*	−1.52 to −1.01
Liminal	−0.18	−0.42–0.05	−0.05	−0.31–0.20	−0.07	−0.32–0.18
Age	0.02	−0.04–0.08	−0.02	−0.08–0.05	−0.01	−0.07–0.05
Gender (F)	0.13	−0.05–0.32	0.12	−0.06–0.31	0.15	−0.04–0.33
CRED			0.26*	0.07–0.45	0.16*	0.05–0.27
% Paedo			−0.03	−0.07–0.00	−0.07	−0.15–0.01
Ex-Cath × CRED			−0.15	−0.38–0.09		
Liminal × CRED			−0.12	−0.40–0.16		
Ex-Cath × Paedo					0.04	−0.06–0.14
Liminal × Paedo					0.05	−0.05–0.15
Observations	247		242		242	
*R*^2^/*R*^2^ adjusted	0.497/0.489		0.535/0.529		0.534/0.518	

*Note:* * confidence interval does not cross 0.

### 3.3. Credibility Enhancing Displays and differences in orthodox Catholic belief between groups

Unsurprisingly, there are large differences in orthodox Catholic belief scores between the three groups. Catholics had a mean score of 5.18 (SD = 1.70), liminal Catholics had a mean score of 3.80 (SD = 1.41), and ex-Catholics had a mean score of 1.52 (SD = 0.94). Ex-Catholics, in other words, largely exhibited outright dismissal of orthodox Catholic beliefs.

**Figure 4.**
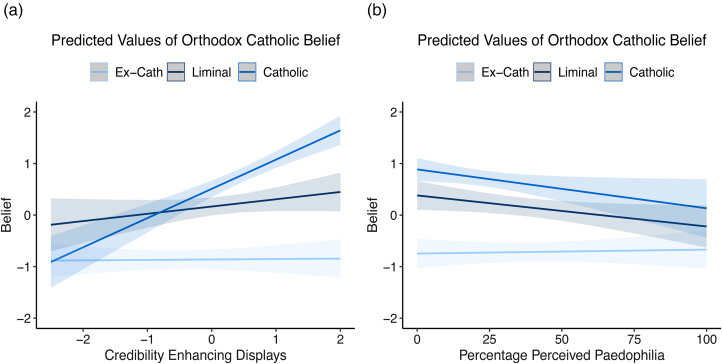
Credibility Enhancing Displays (a) predict orthodox Catholic belief within the Catholic group, but not within the liminal or ex-Catholic groups. No difference is seen in predictions of paedophilia (b). Shaded areas are 95% confidence intervals.

We next examined whether past Credibility Enhancing Display exposure predicted the differences in belief between these groups. Both Credibility Enhancing Displays and clerical paedophilia predictions predict orthodox Catholic belief in models 2 and 3, Credibility Enhancing Displays positively and clerical paedophilia estimates negatively. In model 2, we found that exposure to Credibility Enhancing Displays is predictive of orthodoxy within the Catholic group, but that these effects were reduced or disappeared in the two Church-rejecting groups (Figure 4; simple slopes – Catholic, *ß* = 0.57, 95% CI = 0.41–0.73; liminal, *ß* = 0.14, 95% CI = −0.04–0.32; ex-Catholic, *ß* = 0.03, 95% CI = −0.10–0.16). Model 3 showed no significant difference across groups in effects of paedophilia, although the effects were strongest in the Catholic group and disappeared in the ex-Catholic group (simple slopes – Catholic, *ß* = −0.07, 95% CI = −0.15 to −0.00; liminal, *ß* = −0.06, 95% CI = −0.12 to −0.00; ex-Catholic, *ß* = −0.01, 95% CI = −0.06–0.04).

As can be seen in Figure 4, the interaction effects for ex-Catholics and liminal Catholics show no relationship between level of Credibility Enhancing Displays and orthodoxy for these groups. Thus, past Credibility Enhancing Display exposure predicts whether people will reject the Church, but not the levels of orthodoxy within the two ‘Church-rejecting’ groups (ex-Catholics and liminal Catholics).

### 3.4. Credibility Enhancing Displays and adherence to Catholic morality

Across the groups, we see a similar but smaller difference in adherence to Catholic morality. Catholics had a mean Church moral agreement score of 3.40 (SD = 1.39), liminal Catholics had a mean score of 1.88 (SD = 0.93) and ex-Catholics had a mean score of 1.57 (SD = 1.04).

Next, we ran regression models to see whether Credibility Enhancing Display exposure predicted acceptance of Catholic morality (see Table 3).

**Table 3.** Credibility Enhancing Displays do not predict levels of orthodox Catholic belief in the Church-rejecting groups

	Model 1		Model 2		Model 3	
Predictors	Estimates	95% CI	Estimates	95% CI	Estimates	95% CI
(Intercept)	0.25	−0.14–0.64	0.23	−0.15–0.61	0.21	−0.20–0.62
Ex-Cath	−1.61*	−1.81 to −1.41	−1.32*	−1.54 to −1.11	−1.34*	−1.57 to −1.12
Liminal	−0.60*	−0.81 to −0.39	−0.34*	−0.55 to −0.12	−0.46*	−0.68 to −0.24
Age	0.08*	0.03–0.14	0.02	−0.03–0.07	0.04	−0.02–0.10
Gender (F)	0.12	−0.05–0.29	0.11	−0.05–0.27	0.15	−0.02–0.32
CRED			0.57*	0.41–0.73	0.21*	0.11–0.31
% Paedo			−0.04*	−0.07 to −0.01	−0.07*	−0.15 to −0.00
Ex-Cath × CRED			−0.54*	−0.74 to −0.34		
Liminal × CRED			−0.43*	−0.67 to −0.19		
Ex-Cath × Paedo					0.07	−0.02–0.15
Liminal × Paedo					0.01	−0.08–0.11
Observations	247		242		242	
*R*^2^/*R*^2^ adjusted	0.588/0.581		0.664/0.653		0.627/0.614	

Note: *confidence intervals do not cross 0.

**Table 4.** Credibility Enhancing Displays predict the rejection of conservative Catholic morality in the liminal group

	Model 1		Model 2		Model 3	
Predictors	Estimates	95% CI	Estimates	95% CI	Estimates	95% CI
(Intercept)	0.15	−0.33–0.63	0.18	−0.33–0.68	0.11	−0.41–0.64
Ex-Cath	−1.11*	−1.36 to −0.87	−1.00*	−1.28 to −0.72	−1.03*	−1.32 to −0.74
Liminal	−0.97*	−1.23 to −0.71	−0.84*	−1.12 to −0.55	−0.91*	−1.20 to −0.63
Age	0.13*	0.06–0.19	0.11*	0.04–0.18	0.11*	0.04–0.19
Gender (F)	0.03	−0.18–0.23	−0.02	−0.24–0.19	0.03	−0.19–0.24
CRED			0.30*	0.08–0.52	0.07	−0.06–0.19
% Paedo			0.00	−0.04–0.05	−0.05	−0.15–0.04
Ex-Cath × CRED			−0.26	−0.53–0.01		
Liminal × CRED			−0.47*	−0.79 to −0.16		
Ex-Cath × Paedo					0.08	−0.03–0.19
Liminal × Paedo					0.05	−0.07–0.17
Observations	247		242		242	
*R*^2^/*R*^2^ adjusted	0.383/0.373		0.410/0.390		0.393/0.372	

*Note:* *confidence intervals do not cross 0.

**Figure 5.**
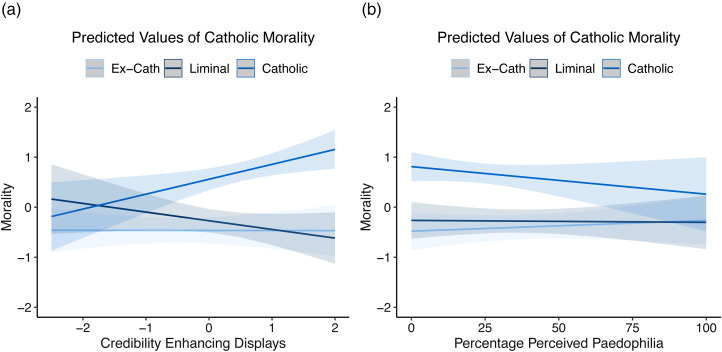
The relationship between past Credibility Enhancing Displays and (a) exposure or paedophilia (b), and endorsement of Catholic moral stances. Shaded areas are 95% confidence intervals.

When we look at the effects of Credibility Enhancing Displays on Catholic morality, we see that there is a significant effect for Catholics (*ß* = 0.30, 95% CI = 0.08–0.52), which disappears in the ex-Catholics (simple slope, *ß* = 0.04, 95% CI = −0.13–0.20) and reverses within the liminal group (simple slope, *ß* = −0.17, 95% CI = −0.42–0.07), and this effect is counter to what one might predict. Liminal Catholics who have been exposed to higher Credibility Enhancing Displays are in fact more likely to *reject* Catholic morality (Figure 5). There is a trend in the opposite direction for those that maintain their affiliation with the Church, although this effect is not quite significant. No significant effects were seen for clerical paedophilia estimates (simple slopes – Catholic, *ß* = −0.05, 95% CI = −0.15–0.04; liminal, *ß* = −0.00, 95% CI = −0.08–0.07; ex-Catholic, *ß* = 0.03, 95% CI = −0.04–0.09).

## 4. Discussion

The data above provide further evidence of the central role of Credibility Enhancing Displays in transmitting religious belief and in sustaining social identities linked to these beliefs, even in those who reject the Catholic Church. Those who were exposed to fewer Credibility Enhancing Displays growing up were more likely to be ex-Catholics, were less likely to believe in God, and among those that remained Catholic, less likely to endorse orthodox Catholic beliefs despite the fact that almost the entire sample were educated in Catholic schools where they would have been regularly exposed to these components of a Catholic worldview. Given the high number of those educated in Catholic schools, where children are exposed to religious discourse (frequently at the hands of press-ganged teachers with little interest in the subject) without much actual practice (Fischer, 2017; Scally, 2021), and given clerical reports that the Catholic education system is essentially impotent at reproducing orthodoxy in the absence of domestic behavioural reinforcement by disengaged ‘culturally Catholic’ parents (Turpin, 2022), religious discourse alone appears to be insufficient to reliably transmit religious belief. The effects of Credibility Enhancing Display exposure on orthodox Catholic morality were less clear.

The data also contain a number of interesting nuances which offer suggestive hints about Irish secularisation. For a start, the perceived prevalence of clerical paedophiles is higher among both groups who have rejected the Church: liminal Catholic and ex-Catholics. This suggests that both groups have responded to clerical abuse scandals by adopting an attitude of moral contempt towards the Church's claims to authority. Despite this, leaving the religion entirely, rather than just rejecting the authority of the Church, is uniquely predicted by lower domestic Credibility Enhancing Display exposure. This may relate to the fact that those with higher Credibility Enhancing Displays will associate Catholicism not just with a media-relayed image of abuse and hypocrisy, but also with potentially more positive experiences of religion drawn from their families. Importantly, the liminal group may reject many of the specific teachings of the Catholic Church (transubstantiation and so forth), but maintains a high level of more general belief in God, unlike those in the ex-Catholic group. Liminal Catholicism may well be a compromise position which reconciles a negative image of the Church with ongoing Catholic affiliation and the maintenance of the central tenet of their religion: belief in God. This provides some evidence that Credibility Enhancing Displays lead to higher maintenance of belief separate from mandated ritual behaviours and strong identification with one's religious institution.

This also supports the two-step process previously suggested for Credibility Enhancing Display-based religious decline. We should expect to see a generation of believers who forgo the costly practices of their religion, but maintain some belief, before we see rapid decline in belief itself (Lanman, 2012; Willard & Cingl, 2017). In Ireland, Mass attendance data and sociological work suggest domestic religious socialisation is in rapid decline. This suggests that the liminal position of disapproval for the Church combined with ongoing Catholic affiliation and maintenance of some religious beliefs may be historically transitory. Those with a lower degree of domestic Credibility Enhancing Display exposure may simply have no commanding alternative associations with Catholicism, and no strongly established belief in God, to push back against the contaminated image of the Church. There may be little reason why they would seek to salvage Catholic affiliation when it is both peripheral and contaminated. Given declines in religious practice and socialisation, this suggests Irish ex-Catholicism and non-religion will grow further in the future.

Credibility Enhancing Displays additionally predict levels of orthodox Catholic belief among Catholics, but less so for the other two groups. For the ex-Catholic group, this is probably unsurprising. Ex-Catholics have probably explicitly rejected Catholic beliefs because these beliefs are symbolic markers of Catholic group identity. We have no data on how strongly they may have held these beliefs prior to their rejection, and thus no data on how much they were influenced by Credibility Enhancing Displays prior to rejecting Catholicism. In other words, becoming ex-Catholic effectively wipes out any signal of past Credibility Enhancing Display effects. It is more difficult to interpret the weaker relationship in the liminal Catholic results. Given their higher perceived prevalence of clerical paedophilia, it is possible that this group represents Catholics who have reacted more strongly to clerical scandals. They remain theists, but they have somewhat disassociated themselves from highly symbolic orthodox tenets that are too closely linked to the (contaminated) authority of the Church. This may be a direct effect of the Church's hypocritical malfeasance on the plausibility of these representations, or it may be a downstream effect of outrage or disgust. Either way would decrease any prior relationship between Credibility Enhancing Displays and orthodox Catholic beliefs (but not Credibility Enhancing Displays and general theism). Thus, high perceived prevalence of the ‘Credibility Undermining Displays’ of clerical paedophilia tends to reduce orthodoxy, but not general theism.

The data on Catholic morality was suggestive. To begin with, the smaller size of the between-group differences here as compared with the orthodox theological beliefs above probably relates to the fact that the Irish population (including Catholics) has in general become more liberal than the Church (e.g. Amarach Research, 2012). We can see that the Catholic group's moral agreement score is considerably lower than their orthodox belief score, and that liminal Catholics are much closer to ex-Catholics on this measure than on the belief measure. One interpretation could be that while ontological beliefs such as the Trinity or the Virgin Birth may appear implausible or bizarre, they are unlikely to provoke negative reactions among more liberalised individuals in the way that conservative moral positions might. Ireland has had prominent referenda on abortion and same-sex marriage that have pitted different normative stances against one another. It has not had socially divisive referenda on whether God is Three Divine Persons in One, or whether Jesus was immaculately conceived.

Interestingly, high Credibility Enhancing Displays in fact predicted the *rejection* of Catholic moral stances among the liminal group. This was an unexpected finding, but there are a number of plausible reasons why it may have been the case. The liminal category is probably a highly varied one, consisting of a range of positions from disengaged cultural Catholics to privatised devout believers who have retained their faith while leaving the Church. This effect within the liminal group might be suggestive of a stronger outrage reaction among those who have more exposure to Credibility Enhancing Displays, and thus should have been more likely to remain within the Church. These high-Credibility Enhancing Display liminals might feel more betrayed by the Church's failure to live up to its own strict standards and thus less willing to endorse strict Catholic morality in turn (see Hilliard, 2003). They may also be liberal ‘deinstitutionalised Catholics’, i.e. devout individuals who have rejected the dominant conservative wing of the Church in favour of a more liberalised and personalised style of Catholicism (Ganiel, 2016, 2019). Their rejection of Catholic morality, a point of considerable social controversy and something that jars with the Church's own moral failings, may serve to project their status as ‘good’ Catholics who do not share the Church's authoritarianism or contamination. In contrast, given the strong cultural and historical links that still prevail between Catholicism and Irish ethnic identity, the low-Credibility Enhancing Display end of the liminal Catholic spectrum might be more representative of religiously disengaged but still socially conservative individuals who retain Catholic group affiliation out of a sense of ethno-national belonging. More research needs to be done before we can be confident in these interpretations.

## Research transparency and reproducibility

Data and analysis code are available in an Open Science Framework repository at: DOI 10.17605/OSF.IO/M3XPH

## Appendix I: Scales

### 1. Catholic belief scale

To what degree do you believe in the following? 0 = do not believe in this at all, and 6 = believe in this completely.

Items:

1. *Transubstantiation: In Mass, the bread and wine really become the flesh and blood of Jesus*
2. *Papal Infallibility: what the Pope says is always correct*
3. *Resurrection: Jesus came back from the dead*
4. *Miracles: the miracles of the saints are real*
5. *The Virgin Birth: Mary was a virgin when she gave birth to Jesus*
6. *Confession: Priests have the power from God to forgive our sins.*

### 2. Catholic Church moral agreement scale

To what extent do you agree or disagree with the Catholic Church's position on the following issues? ‘0’ = Strongly disagree with the Church, and ‘6’ = strongly agree with the Church

Items:

1. *Abortion*
2. *Same-sex marriage*
3. *Divorce*
4. *Sex before marriage*
5. *Priestly celibacy (priests can't marry)*
6. *Female priests*
7. *Contraception*

### 3. Catholic CRED scale

Instructions: The following questions ask about experiences during your upbringing that relate to Catholicism. Specifically, the questions ask about your perceptions of your primary caregiver or caregivers (i.e. parents or guardians). Please answer each of the following according to your overall impression of your caregiver(s) on the following scale: 0 = to no extent at all … 6 = to an extreme extent.

Items:

1. *To what extent did your caregiver(s) attend Catholic services (such as Mass) or other Catholic gatherings?*
2. *To what extent did your caregiver(s) engage in Catholic volunteer or charity work?*
3. *Overall, to what extent did your caregiver(s) act as good Catholic role models?*
4. *Overall, to what extent did your caregiver(s) make personal sacrifices to Catholicism?*
5. *To what extent did your caregiver(s) act fairly to others because their Catholic religion taught them so?*
6. *To what extent did your caregiver(s) live a religiously pure life according to the rules of the Catholic Church?*
7. *To what extent did your caregiver(s) avoid harming others because their Catholicism taught them this?*
8. *To what extent did your caregiver(s) perform Catholic prayers at home (such as saying the rosary, etc.)?*

## References

[ref1] Amarach Research (2012). Contemporary Catholic Perspectives (Survey commissioned by the Association of Catholic Priests). https://www.associationofcatholicpriests.ie/wp-content/uploads/2012/04/Contemporary-Catholic-Perspectives.pdf (accessed 25 July 2015).

[ref2] Angelides, S. (2005). The emergence of the paedophile in the late 20th century. Australian Historical Studies, 36, 272–295.

[ref3] Atran, S. (2010). Talking to the enemy: Violent extremism, sacred values, and what it means to be human. Allen Lane/Penguin.

[ref4] Boyd, R., Richerson, P. J., & Henrich, J. (2011). The cultural niche: Why social learning is essential for human adaptation. Proceedings of the National Academy of Sciences of the United States of America, 108(Suppl 2), 10918–10925. 21690340 10.1073/pnas.1100290108 PMC3131818

[ref5] Bryan, J. H., & Walbek, N. H. (1970). Preaching and practicing generosity: Children's actions and reactions. Child Development, 41(2), 329–353.

[ref6] Bullivant, S. (2017). Religion in Ireland: Recent trends and possible futures. Presentation delivered to the Iona Institute, Dublin, 24 August.

[ref7] Day, A. (2011). Believing in belonging: Belief and social identity in the modern world. Oxford University Press.

[ref8] Donnelly, S., & Inglis, T. (2010). The media and the Catholic Church in Ireland: Reporting clerical child sex abuse. Journal of Contemporary Religion, 25(1), 1–19.

[ref9] Egan, K. (2011). Remaining a Catholic after the Murphy Report. Columba.

[ref10] Eriksen, T. H., Bal, E. W., & Salemink, O. H. J. M. (Eds.) (2010). A world of insecurity: Anthropological perspectives on Human Security. Anthropology, Culture and Society. Pluto Press.

[ref11] Finnegan, F. (2001). Do penance or perish: Magdalen asylums in Ireland. Oxford University Press.

[ref12] Fischer, K. (2017). Separate but equal? Schools and the politics of religion and diversity in the Republic of Ireland. Manchester University Press.

[ref13] Fuller, L. (2002). Irish Catholicism since 1950: The undoing of a culture. Gill & MacMillan.

[ref14] Ganiel, G. (2016). Transforming Post-Catholic Ireland: Religious practice in late modernity. Oxford University Press.

[ref15] Ganiel, G. (2019). Religious practice in a Post-Catholic Ireland: Towards a concept of ‘extra-institutional religion’. Social Compass, 66(4), 471–487.

[ref16] Garvin, T. (2004). Preventing the future: Why was Ireland so poor for so long. Gill Books.

[ref17] Gervais, M., & Fessler, D. (2017). On the deep structure of social affect: Attitudes, emotions, sentiments, and the case of ‘contempt’. Behavioral and Brain Sciences, 39, 1–77. 10.1017/S0140525X16000352 27001168

[ref18] Gervais, W. M., Najle, M. B., & Caluori, N. (2021). The origins of religious disbelief: A dual inheritance approach. Social Psychological and Personality Science, 12(7), 1369–1379.

[ref19] Gervais, W. M., Willard, A., Norenzayan, A., & Henrich, J. (2011). The cultural transmission of faith: Why innate intuitions are necessary, but insufficient, to explain religious belief. Religion, 41, 389–410.

[ref20] Goode, H., McGee, H., & O'Boyle, C. (2003). Time to listen: Confronting child sexual abuse by Catholic clergy in Ireland. The Liffey Press.

[ref21] Greenberg, J., Solomon, S., & Pyszczynski, T. (1997). Terror management theory of self esteem and cultural worldviews: Empirical assessments and conceptual refinements. Advances in Experimental Social Psychology, 12, 417–433.

[ref22] Grusec, J. E. (1971). Power and the internalization of self-denial. Child Development, 42(1), 93–105.

[ref23] Harper, L., & Sanders, K. M. (1975). The effect of adults’ eating on young children's acceptance of unfamiliar foods. Journal of Experimental Child Psychology, 20, 206–214.

[ref24] Harris, P., Pasquini, E. S., Duke, S., Asscher, J. J., & Pons, F. (2006). Germs and angels: The role of testimony in young children's ontology. Developmental Science, 9(1), 76–96. 16445398 10.1111/j.1467-7687.2005.00465.x

[ref25] Henrich, J. (2009). The evolution of costly displays, cooperation, and religion: Credibility enhancing displays and their implications for cultural evolution. Evolution and Human Behaviour, 30, 244–260.

[ref26] Henrich, J. (2015). The secret of our success: How culture is driving human evolution, domesticating our species, and making us smarter. Princeton University Press.

[ref27] Hilliard, B. (2003). The Catholic Church and married women's sexuality: Habitus change in the late 20th century Ireland. Irish Journal of Sociology, 12(2), 28–49.

[ref28] Hogan, C. (2019). Republic of shame: Stories from Ireland's institutions for ‘fallen’ women. Penguin.

[ref29] Inglehart, R. (2021). Religion's sudden decline: What's causing it, and what comes next? Oxford University Press.

[ref30] Inglis, T. (1998). Moral monopoly: The rise and fall of the Catholic Church in modern Ireland. University College Dublin.

[ref31] Inglis, T. (2014). Meanings of life in contemporary Ireland: Webs of significance. Routledge.

[ref32] Iona Institute (2011). Attitudes towards the Catholic Church. Amarach Research.

[ref33] Jordan, J. J., Sommers, R., Bloom, P., & Rand, D. G. (2017). Why do we hate hypocrites? Evidence for a theory of false signalling. Psychological Science, 2017(1), 1–13. 10.1177/0956797616685771 28107103

[ref34] Keenan, M. (2012). Child sexual abuse and the Catholic Church: Gender, power and organisational culture. Oxford University Press.

[ref35] Kraft-Todd, G. T., Bollinger, B., Gillingham, K., Lamp, S., Rand, D. G. (2018). Credibility-enhancing displays promote the provision of non-normative public goods. Nature, 563, 245–248. 30356217 10.1038/s41586-018-0647-4

[ref36] Langston, J., Speed, D., & Coleman III, T. J. (2020). Predicting age of atheism: Credibility enhancing displays and religious importance, choice, and conflict in family of upbringing. Religion, Brain & Behavior, 10(1), 49–67.

[ref37] Lanman, J. (2012). The importance of religious displays for belief acquisition and secularisation. Journal of Contemporary Religion, 27(1), 49–65.

[ref38] Lanman, J. A., & Buhrmeister, M., (2016). Religious actions speak louder than words: Exposure to CREDs predicts theism. Religion, Brain and Behavior, 7(1), 3–16.

[ref39] Larkin, E. J. (1976). The historical dimensions of Irish Catholicism. CUA Press.

[ref40] Maij, D. L. R., van Harreveld, F., Gervais, W., Schrag, Y., Mohr, C., & van Elk, M. (2017). Mentalizing skills do not differentiate believers from non-believers, but credibility enhancing displays do. PLoS ONE, 12(8), e0182764. 28832606 10.1371/journal.pone.0182764 PMC5568287

[ref41] McWilliams, D. (2019). Renaissance nation: How the Pope's children rewrote the rules for Ireland. Gill & MacMillan.

[ref42] Navarrete, C., & Fessler, D. (2005). Normative bias and adaptive challenges: A relational approach to coalitional psychology and a critique of terror management theory. Evolutionary Psychology, 3, 297–325.

[ref43] Norris, P., & Inglehart, R. (2011). Sacred and secular: Religion and politics worldwide, 2nd ed. Cambridge.

[ref44] O'Toole, F. (2021). We don't know ourselves: A personal history of Ireland since 1958. Head of Zeus.

[ref45] Penet, J. (2008). From idealised moral community to real Tiger society. *The Catholic Church in secular Ireland.* Estudios Irlandeses, 3, 143–153.

[ref46] Pine, E. (2011). The politics of Irish memory: Performing remembrance in contemporary Irish Culture. Palgrave Macmillan.

[ref47] Raftery, M., & O'Sullivan, E. (2009). Suffer the little children: The inside story of Ireland's industrial schools. New Island Books.

[ref48] Ribberink, E., Achterberg, P., & Houtman, D. (2013). Deprivatization of disbelief?: Non-religiosity and anti-religiosity in 14 western European countries. Politics and Religion, 6, 101–120.

[ref49] Scally, D. (2021). The best Catholics in the world: The Irish, the Church, and the end of a special relationship. Penguin Books.

[ref50] Shupe, A. (1997). Vicissitudes of public legitimacy for religious groups: A comparison of the Unification and Roman Catholic churches. Review of Religious Research, 39(2), 172–183.

[ref51] Sosis, R., Kress, H. C., & Boster, J. S. (2007). Scars for war: Evaluating alternative signalling explanations for cross-cultural variance in ritual costs. Evolution and Human Behaviour, 28(4), 234–247.

[ref52] Turpin, H. (2019). Leaving Roman Catholicism. In The handbook of leaving religion (pp. 186–199). Brill.

[ref53] Turpin, H. (2022). Unholy Catholic Ireland: Religious hypocrisy, secular morality, and Irish irreligion. Stanford University Press.

[ref54] Turpin, H., Andersen, M., & Lanman, J. A. (2017). CREDs, CRUDs, and Catholic scandals: Experimentally examining the effects of religious paragon behavior on co-religionist belief. Religion, Brain & Behavior, 87, 1–13.

[ref55] Walster, E., Aronson, E., & Abrahams, D. (1966). On increasing the persuasiveness of a low prestige communicator. Journal of Experimental Social Psychology, 2, 325–342.

[ref56] Willard, A. K., & Cingl, L., (2017). Testing theories of secularization and religious belief in the Czech Republic and Slovakia. Evolution and Human Behavior, 38(5), 604–615.

[ref57] Willard, A. K., Henrich, J., & Norenzayan, A. (2016). Memory and belief in the transmission of counterintuitive content. Human Nature, 27(3), 221–243. 27100109 10.1007/s12110-016-9259-6

[ref58] Win-Gallup (2012). International index of religion and atheism. https://sidmennt.is/wp-content/uploads/Gallup-International-um-tr%C3%BA-og-tr%C3%BAleysi-2012.pdf (accessed 4 October 2015).

[ref59] Wollschleger, J., & Beach, L. (2011). A cucumber for a cow: A theoretical explanation of the causes and consequences of religious hypocrisy. Rationality and Society, 23(2), 155–174.

